# Cancer epidemiology literature from India: Does it reflect the reality?

**DOI:** 10.1093/pubmed/fdz160

**Published:** 2019-12-27

**Authors:** K V Deepa, Jubina Balan Venghateri, Monty Khajanchi, Anita Gadgil, Nobhojit Roy

**Affiliations:** 1 Manipal Hospital, Delhi, India; 2 WHO Collaborating Centre for Research in Surgical Care Delivery in LMIC, Mumbai, India; 3 Department of Surgery, K.E.M. Hospital, Mumbai, India; 4 Department of Surgery, WHO Collaborating Centre for Research in Surgical Care Delivery in LMIC, BARC Hospital, Mumbai, India; 5 Department of Public Health Sciences, Karolinska Institute, Stockholm, Sweden

**Keywords:** cancer, epidemiology, incidence, India, risk factors

## Abstract

**Background:**

The alarming escalation of cancers over infectious diseases in the lower and middle-income countries warrants a better understanding of this epidemiological transition. The epidemiology of cancers in India is sparsely addressed in the literature. Hence, in this manuscript, we present the review done, on research manuscripts, addressing cancer incidence, trends and risk factors from India over the last 12 years. Studies addressing screening, treatment and clinical trials were excluded.

**Methods:**

We evaluated the studies for the theme addressed, study design, sample size, the region of origin and whether it was population or hospital-based study.

**Results:**

The studies highlighted a significant shortage of multicenter population-based data in the incidence and risk factors associated with various malignancies in India. Further, we also observed that there was a relative lack of information from the northern and northeastern parts of India. The reviewed articles also indicated the need for a robust design for the studies, large sample size and uniformity in reporting incidence for appropriately drawing conclusions from a study. Reporting of country-specific risk factors with their geographical variations was also sparse.

**Conclusion:**

Overall, the cancer epidemiology literature from India is sparse. More studies with robust designs representing all parts of the country are currently needed.

## Introduction

The low- and middle-income countries (LMICs) are undergoing an epidemiological transition, wherein the burden of communicable diseases is declining and non-communicable diseases like cancers are on the rise.^1^ An estimation of 20 million cancer cases is expected in LMICs by 2025.^2^ There has been a considerable variation in the incidence of cancers between high-income countries (HICs) and LMICs.^3^ The incidence varies from 95/10 000 in the LMICs to over 571/100 000 in the HIC countries in both men and women.^4^ The situation is dramatically altering in LMICs over last few decades especially due to the lifestyle changes, industrialization, migration of population from rural areas to cities and increased life expectancy.^5^^,^^6^ Indian scenario in cancer incidence shows similar upwards trends. The country also shows great variation in the incidence and epidemiology of all cancers, especially stomach, esophageal and breast cancers, across its span, due to variation in socio-cultural practices and lifestyle differences.^7^ The literature on the epidemiology of cancers in India is sparse. Mallath et al.^7^ in their series of articles on cancer scenario in India have partly addressed this issue, yet a detailed systematic review of geographical distribution, quality of the studies published and issues addressed is lacking. Studies that bring out the incidence trends and patterns of cancers and epidemiology are crucial, as they will guide in planning infrastructure, improving treatment facilities, assist in strengthening diagnostic procedures and implementing necessary screening programs for a wide variety of cancers in future.^8^ As India prepares for a massive screening of Non Communicable Diseases (NCD), including cancer in the ‘Ayushman Bharat’ (PM-JAY) scheme, it is imperative that we know the current trends and burden of cancers (denominator) in India, to plan feasible cancer care in India. This review aims at evaluating the larger picture of cancer epidemiology literature in India. We reviewed the literature published so far in the field of cancer epidemiology, highlighted the lacunae in it and provided possible pointers for future direction.

## Materials and methods

**Fig. 1:**
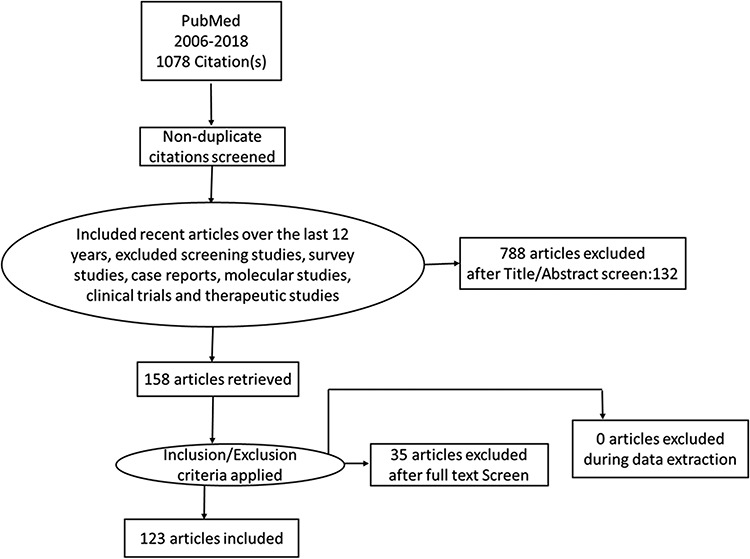
Process for selection of manuscripts

We checked the list of journals included in PubMed to ensure articles in all leading Indian journals published in English were included. The search was done by using ‘India’[all Fields] AND (‘Neoplasms’[Mesh Terms]) AND (‘Prevalence’[MeSH Terms] OR ‘Incidence’[MeSH Terms] OR ‘Cost-benefit analysis’[MeSH Terms]) NOT ‘Therapy’ NOT ‘Molecular’ NOT ‘genetics’ NOT ‘Screening’ NOT ‘case reports’ NOT ‘Clinical Trials’. We limited studies to those that were published between 2006 and 2018 because the increase in NCDI in the country is relatively recently being reported because of a series of policy changes, such as decentralized planning under the National Rural Health Mission in 2005. The review was limited to peer-reviewed articles published in English and excluded systematic reviews, narrative reviews, study protocols, reports, opinions, editorials, letters to the editor and commentaries. Clinical trials and studies describing or comparing therapies were also excluded to narrow the search. The articles that were not relevant to the topic were excluded by reading the abstracts of the articles and then the remaining studies were shortlisted for a detailed review. The review was registered on PROSPERO register, Reg. number: CRD42017058579. (http://www.crd.york.ac.uk/PROSPERO). A PRISMA 2009 checklist was applied to the review and writing process.^9^ Two independent reviewers reviewed the manuscripts and disagreements in the assessment were resolved by discussions and meetings. The third reviewer randomly selected manuscripts from the final selected manuscripts and analyzed them separately using the same criteria as the first two reviewers. The review of the articles by all the three reviewers was matched and agreement was reached between three reviewers. Discrepancies between the reviewers were resolved by strictly adhering to the criteria and discussions. The pre-designated review criteria were sample size, level of evidence, whether the data were primary or secondary, whether the study was community or hospital-based, multicentric or single-center data and whether the project was funded or unfunded were also looked into. We looked at the primary theme addressed by the manuscripts reviewed. These were then stratified into manuscripts addressing incidence trends or prevalence, regional variations, risk factors and associated conditions. Calculations and computations based on population-based registry data were considered as a secondary source for the data, whereas hospital-based registry data were considered data from the primary source. Figure 1 shows the exclusion process to narrow the detailed review to 123 studies.

## Results

A total of 123 manuscripts were found eligible for the final analyses. About 94 studies reported primary data analysis and 64 studies (52%) presented a single-center data mainly from the tertiary care cancer institutes situated in metropolitan cities in India. Major contributors from tertiary care centers were, All India Institute of Medical Sciences (AIIMS, New Delhi); Tata Memorial Hospital (TMH), Mumbai; Vellore Medical College, Bangalore; National Cancer Registry Program, Sanjay Gandhi Post Graduate Institute, Lucknow; Post Graduate Institute Chandigarh; Karnataka Institute of Medical Sciences; IMS Karnataka; and B. Borooah Guwahati. About 29 (23.5%) studies analyzed secondary data from population-based cancer registries (PBCRs) under the National Cancer Registry Program by Government of India. Primary multicentric and population-based primary data were presented by 8 and 11 studies, respectively. Table 1 shows the characteristics of the studies and their distribution.

**Table 1:** Characteristics of the studies

*Characteristic of the study*	*N = 123 (%)*
Level of evidence	
II	39 (31.7)
III	57 (46.3)
IV	18 (14.6)
V	9 (7.3)
Study designs	
Cross-sectional	46 (37.4)
Time trends and mathematical modeling	28 (22.8)
Cohort studies	23 (18.7)
Case control	15 (12.2)
Case series	7 (5.7)
Review	2 (1.6)
Meta-analysis	1 (0.8)
Research communications	1 (0.8)
Data source type	
Primary	94 (76.4)
Secondary	29 (23.6)
Sample size	
<100	16 (13)
100–1000	42 (34.14)
1000–10 000	23 (18.7)
>10 000 (includes PBCR secondary data)	42 (34.17)
Issues discussed in the papers	
Epidemiology and trends of incidences	82 (66.66)
Risk factors	36 (44%)
Comorbid conditions associated with cancers	5 (6.5%)
Cancers addressed	
Breast and female reproductive tract	18 (15.4)
Tobacco-related cancers of head, neck and upper aerodigestive tract including lung	27
Leukemia and lymphoma	9 (7.3)
Brain cancer	5 (4.06)
Gastrointestinal cancers	17 (13.8)
Kidney and prostate cancers	5 (3.25)
Others including thyroid and skin cancers	4 (0.8)
All cancers	33 (26.8)
Childhood cancers	5 (4.06)

### Geographical distribution of studies

**Fig. 2:**
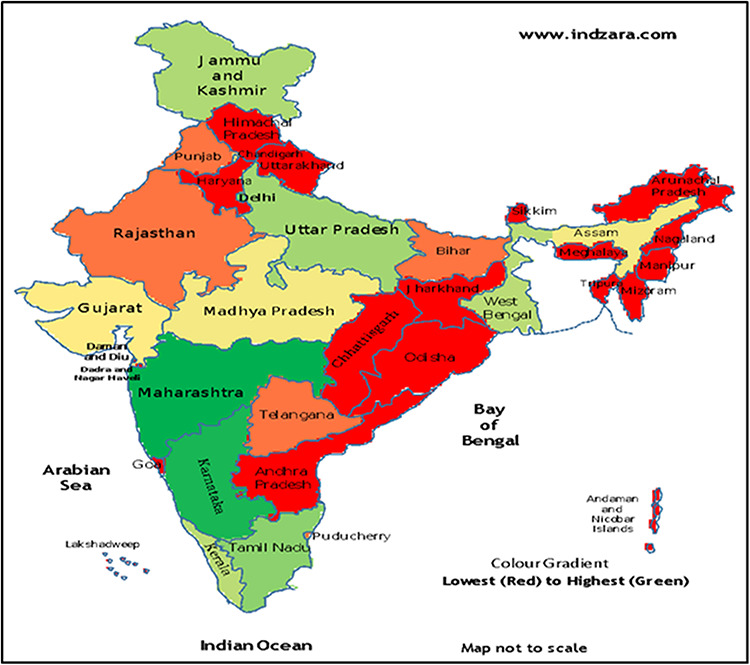
Map depicting the distribution of cancer trends and risk factor-related manuscripts from various states in India.

The analysis using 123 articles reviewed showed that the information of epidemiology of cancers was lacking from eastern, northern and northeastern parts of the country (Fig. 2). Southern states contributed 28 (22%) articles, whereas the northeastern states including the metropolis of Kolkata contributed 9 (7.3%) articles.

### Cancer trends

Two-thirds of the papers discussed Cancer incidences and trends within the epidemiology literature (*n* = 82, 66%). The primary data on cancer incidence (*n* = 64, 52%) were mainly hospital-based and reported from the proportion of cancer patients seen in various departments in the hospital and expressed as percentages. The hospital-based data did not report age-standardized incidence (ASR) or temporal trends. The studies analyzing the secondary data from the population-based registries reported results in age-standardized rates and annual percentage changes or trends of incidence. Breast and cervical cancers, followed by tobacco-related cancers generated the highest number of research papers (18 and 22 respectively), among all the cancers. Prostate and childhood cancers remained largely unaddressed with three and five studies addressing them respectively. Commonest cancers in India emerged from this review were breast and cervix in females and tobacco-related cancers in the males. The maximum rise in the incidence of cancers was seen in the urban registries of Delhi and Mumbai and the maximum increase was noted in breast cancer. Eleven of the 22 studies that addressed cancer trends have documented breast cancer incidence trend. Lung, esophagus, lip and oral cancers were three top cancers reported in men, in this review.

### Risk factors

Thirty-six (29%) studies have addressed the risk factors for various cancers. The main cancers where risk factors were studied were gastrointestinal cancers (*n* = 11, esophageal, gallbladder, liver, colorectal, stomach) followed by cancers affecting head, face and neck (*n* = 8) and breast cancer (*n* = 5). Five studies addressed risk factors for multiple cancers. Three studies looked into risk factors of genitourinary cancers in both males and females. Nineteen (15.4%) manuscripts looked into the effects of tobacco in its various forms as a risk factor, followed by diet (*n* = 8) and alcohol (*n* = 6). Seven of the 19 manuscripts, describing tobacco as a risk factor, have studied the tobacco in smokeless tobacco form. Chemicals and medicines were the other common risk factors that were studied in the development of cancers.

### Other characteristics and limitations of the manuscripts

Fifty (41%) of the reviewed papers addressed the limitations of the methodology and data. Fifty (41%) studies addressed the confounding factors while assessing results and have been accounted for it. Of the 123 articles reviewed here, only 25% (31 articles) reported the funding agency. The source of funding was not mentioned for three-fourths of articles.

## Discussion

In this review, our main finding was that the cancer epidemiological data from India largely were hospital-based, single-center data from tertiary care hospitals or PBCRs. We also found that community-based studies were few, and there was paucity of primary multicenter data. We observed that breast, cervix and tobacco-related cancers were majorly reported. Further, we also report that some regions of the country were better represented than others.

The literature reviews about peer-reviewed published epidemiological data on cancers in India, addressing the existing picture and gaps are sparse.

This study adds the information about the characteristics of the studies available, the cancers addressed and their incidence and trends as seen through this published literature and the risk factors described along with the areas and states that are depicted through published manuscripts.

### Study characteristics

Analyzing the characteristics of the manuscripts reviewed, it was seen that tertiary care centers and regional cancer centers generated most of the published single-center data. This represents the population burden poorly, as there exists a selection bias as it may not include the patients who could not reach these tertiary care facilities due to lack of access or affordability. In addition, Data on incidence was not age standardized to be able to compare with the data from the rest of the country or world, which is a limitation. Documents on cancer registries mention that hospital-based cancer registries cannot be used for policy decisions or planning for the same reason of selection bias due to poor access to the healthcare facilities.^10^ Hence, primary population-based data of incidence are needed to represent the true nature and magnitude of the cancer burden. PBCRs are relatively new in India compared to the developed world. Only 10% of the population is covered by various cancer registries in India.^11^ This raises a concern about whether the PBCR data really represent the Indian population but is the best information available in the current scenario.^12^ PBCR is a program of The Indian Council of Medical Research (ICMR) and is a resource-intensive process. Hence, there is a paucity of cancer registries representing rural and remote areas. The program yet needs to develop and cover more districts and states to be able to show a true representation. The burden of cancers, trends, mathematical modeling for predictions and descriptive epidemiology has been the main outputs of the PBCR based studies.^12^ Two-thirds of the articles used sample size less than 10 000. Conducting a primary data collection is labor-intensive and costly process, and this could have led to limited studies recruiting larger samples and multicentric data as reflected in our literature review.^13^^,^^14^ Most of the primary data collection involved a single institution experience with small sample size, compared to the western cancer literature involving multicentric large sample studies.^15^^,^^16^ This may give a skewed picture as the number of cases diagnosed and treated would depend on the expertise and experience of the individual investigator. Ethically sensitive data collection could be a problem in areas where literacy is low and cultural barriers may not allow participation.^10^ A large number of the tertiary centers published the cross-sectional studies for ease of design and low need for resources as described in our study.^17^^,^^18^

### Incidence and trends

Seventy-six papers studied incidence and trends. There was no uniformity in reporting the incidence rates. The population-based registries and trends from secondary data have reported the incidence as ASR and expected annual percentage change. The hospital-based studies have represented incidence as a percentage of patients treated. The non-uniformity in reporting and predominance of hospital-based registries lead to misrepresentation of the incidence of cancers. Keeping in line with the changing epidemiological picture in LMICs, the cancer incidence rise was reported in all of our studied manuscripts.^1^

### Risk factors

The most common risk factor studied is tobacco in the form of smoking and the smokeless, which is most commonly seen in the Indian subcontinent.^19^ The cancers studied with tobacco as a risk factor were gastrointestinal cancers, head and neck cancers, lung cancers, prostate cancer and urinary tract cancers. The use of tobacco in its various forms is estimated to be a risk factor in 45% male cancers and 17% female cancers in India.^20^^,^^21^ This keeping in line with the most prevalent cancers among males. India needs to have more studies addressing risk factors as the etiology may vary considerably between the geographic location, diet, addiction and different cultural practices. A good example of cultural practices affecting risk factor influence is the association of human papilloma virus in oral cancer. This is a major risk factor for oral cancers in the world, but it may not hold true in the Indian scenario and Indian study has shown no similar association of human papillomavirus with oral/hypopharyngeal cancers.^22^ Cancer cervix is the second most common cancer-affecting females in India. However, only two studies highlight the risk factors for this cancer in India.^23^ The proportion of prevalence to publications was largely maintained in all cancers while studying the incidences. We found that the studies addressing risk factors are not proportionately large in numbers compared to prevalence. Studies on Tobacco re- lated cancers are highest in numbers, proportionate to their prevalence but cervix and breast cancers yet need to be explored for their risk factors as the number of studies for modifiable risk factors for these two cancers are low. The design of the risk factor studies reviewed lacks any randomized control trials (RCT) and large prospective cohorts. This may be due to the complexity of conducting RCT and high costs. Most of the studies addressing risk factors have incorporated cross-sectional or case–control study designs for their studies.

### Funding sources for studies

Of the 123 articles reviewed here, very few studies reported their source of funding from either government or private-based funding institutions. Majority of the studies did not report the source of funding. The International Committee of Medical Journal Editors in 2001 had reinforced and endorsed the requirement of disclosure of funding source/s for studies based on the conception, design, data collection, analysis and publication role of the funding source/s and the authors involved in the study.^24^ Further, New England Journal of Medicine had also revised its rules pertaining to conflict of interest.^25^ It is not clear whether the lack of significant funding or potential conflict of interest had resulted in either withholding or no disclosure of the funding source. Nevertheless, authors and editors should be encouraged to adhere to the guidelines and voluntarily disclose the funding source of any study.

This study tries to highlight what is available and what is needed in the epidemiological studies on cancers in India; however, it has some limitations. We have included manuscripts published in pubmed only, and other databases are not included. We may have also missed some of the data published as government reports and gazettes.

## Conclusion

To conclude, the authors found a paucity of primary, multicenter, high evidence level data from the community in the field of incidence as well as risk factors of cancers. Certain geographic regions were poorly represented and the existing studies suffered poor design, low sample size and selection biases. This highlights the urgent need for funding cancer epidemiological studies that can lead the planning and prioritization for better cancer control, in India.

## Notes

**K.V. Deepa**, Consultant Surgeon

**Jubina Balan Venghateri**, Consultant WHOCC

**Monty Khajanchi **, Assistant Professor

**Anita Gadgil**, Head, Department of Surgery

**Nobhojit Roy**, Affiliate, Consultant
